# Potentially Avoidable Hospitalizations Among Historically Marginalized Nursing Home Residents

**DOI:** 10.1001/jamanetworkopen.2024.9312

**Published:** 2024-05-02

**Authors:** Leah V. Estrada, Veronica Barcelona, Lara Dhingra, José A. Luchsinger, Andrew W. Dick, Laurent G. Glance, Patricia W. Stone

**Affiliations:** 1 Brookdale Department of Geriatrics and Palliative Medicine, Icahn School of Medicine at Mount Sinai, New York, New York; 2 Columbia University School of Nursing, New York, New York; 3 MJHS Institute for Innovation in Palliative Care, New York, New York; 4 Department of Family and Social Medicine, Albert Einstein College of Medicine, Bronx, New York; 5 Departments of Medicine and Epidemiology, Columbia University Irving Medical Center; 6 RAND Corporation, Boston, Massachusetts; 7 Department of Anesthesiology and Perioperative Medicine, University of Rochester Medical Center, Rochester, New York

## Abstract

**Importance:**

Nursing home (NH) transfers to hospitals are common and have been associated with cognitive decline; approximately 45% of NH hospital transfers are potentially avoidable hospitalizations (PAHs).

**Objective:**

To determine PAH incidence for historically marginalized NH residents with severe cognitive impairment compared with non-Hispanic White residents.

**Design, Setting, and Participants:**

This cross-sectional study merged 2018 Centers for Medicaid & Medicare Services datasets and LTCFocus, a public dataset on US NH care, for US NH residents aged 65 years and older who had a hospitalization. Analyses were performed from January to May 2022.

**Exposure:**

Race and ethnicity of NH residents.

**Main Outcomes and Measures:**

Racial and ethnic differences in resident-level annual rates of PAHs were estimated for residents with and without severe cognitive impairment (measured using the Cognitive Function Scale), controlling for resident characteristics, comorbidities, dual eligibility, and time at risk. PAHs were defined as NH hospital transfers that resulted from neglectful NH care or for which NH treatment would have been appropriate.

**Results:**

Of 2 098 385 NH residents nationwide included in the study, 7151 (0.3%) were American Indian or Alaska Native, 39 873 (1.9%) were Asian, 229 112 (10.9%) were Black or African American, 99 304 (4.7%) were Hispanic, 2785 (0.1%) were Native Hawaiian or Pacific Islander, 1 713 670 (81.7%) were White, and 6490 (0.3%) were multiracial; 1 355 143 (64.6%) were female; 128 997 (6.2%) were severely cognitively impaired; and the mean (SD) age was 81.8 (8.7) years. PAH incidence rate ratios (IRRs) were significantly greater for residents with severe cognitive impairment compared with those without. In unadjusted analyses comparing historically marginalized residents with severe cognitive impairment vs non-Hispanic White residents with severe cognitive impairment, American Indian or Alaska Native residents had a 49% higher PAH incidence (IRR, 1.49 [95% CI, 1.10-2.01]), Black or African American residents had a 64% higher incidence (IRR, 1.64 [95% CI, 1.48-1.81]), and Hispanic residents had a 45% higher incidence (IRR, 1.45 [95% CI, 1.29-1.62]). Higher incidences persisted for historically marginalized residents with severe cognitive impairment vs non-Hispanic White residents with severe cognitive impairment in adjusted analyses. Asian residents had a 24% higher PAH incidence (IRR, 1.24 [95% CI, 1.06-1.45]), Black or African American residents had a 48% higher incidence (IRR, 1.48 [95% CI, 1.36-1.60]), and Hispanic residents had a 27% higher incidence (IRR, 1.27 [95% CI, 1.16-1.39]).

**Conclusions and Relevance:**

In this cross-sectional study of PAHs, compared with non-Hispanic White NH residents, historically marginalized residents had increased PAH incidence. In the presence of severe cognitive impairment, incidence rates increased significantly compared with rates for residents without severe cognitive impairment. These results suggest that identification of residents with severe cognitive impairment and proper NH care may help prevent further cognitive decline by avoiding PAHs.

## Key Points

**Question:**

What is the potentially avoidable hospitalization incidence rate among historically marginalized nursing home residents with and without severe cognitive impairment?

**Findings:**

This cross-sectional study of 2 098 385 nursing home residents found that Black or African American and Hispanic nursing home residents with severe cognitive impairment experienced the highest incidence of potentially avoidable hospitalizations.

**Meaning:**

These results suggest that efforts to reduce potentially preventable hospitalizations among nursing home residents should target Black or African American and Hispanic residents, especially those with severe cognitive impairment approaching the end of life.

## Teaser

This cross-sectional study investigates PAH incidence for historically marginalized NH residents with severe cognitive impairment compared with non-Hispanic White residents.

## Introduction

An estimated 70% of all people with severe cognitive impairment die in nursing homes (NHs), and more than half of NH residents have cognitive impairment.^1^ Annually, 1 in 4 NH residents are hospitalized, which is associated with decline in cognitive function.^2^ Cognitive decline can progress from severe disability to dementia and ultimately death.^3^

Across the US, Black or African American older adults have the highest prevalence of severe cognitive impairment, followed by Hispanic older adults.^4^ Severe cognitive impairment typically indicates the individual is approaching the end of life (EOL) and is characterized by decline in function, purposeful movements, language abilities, and ability to recognize people.^4^ Notably, race and ethnicity are social constructs, not biological variables, and therefore a proxy measure of racism.^5^ Historically marginalized residents (ie, American Indian or Alaska Native, Asian, Black or African American, Hispanic, Native Hawaiian, and Pacific Islanders) often enter the NH in poorer health and with more comorbidities than White residents, related to generational cycles of poverty, mistrust of health care practitioners and the health care systems, and experiences of racism and discrimination that have led to delayed care.^6,7^ Historically marginalized NH residents also have higher rates of hospitalizations overall.^8,9,10,11,12,13^ EOL care for individuals with severe cognitive impairment should be aligned with their goals of care, offered to ease symptom burden, and improve quality of life, which hospitalizations do not always do.

Recent national initiatives have aimed to reduce NH hospitalizations and identify those that are potentially avoidable (eg, dehydration, pressure injuries, and asthma).^14,15,16^ Potentially avoidable hospitalizations (PAHs) result from neglectful NH care or that which NH treatment would have been appropriate.^17^ Despite pervasive racial and ethnic inequities in NH care (eg, segregation and NHs with poor outcomes,^18^ worse EOL care,^19^ fewer palliative care services^20^), limited knowledge exists about PAH racial and ethnic inequities in patients with severe cognitive impairment. The study objective was to identify racial and ethnic incidence rates for PAHs among all NH residents, with and without severe cognitive impairment, who are approaching the EOL.

## Methods

### Study Design, Setting, and Participants

The Columbia University institutional review board approved this cross-sectional study and waived the need for informed consent because the use of secondary data was not deemed human participants research. The Strengthening the Reporting of Observational Studies in Epidemiology (STROBE) reporting guideline was followed. The study sample included all NH residents with a long stay (>100 days) and aged 65 years and older who resided in an NH in the US in 2018 and experienced a hospitalization. Analyses were performed from January to May 2022.

### Data Sources

Four national datasets from 2018 were merged for this analysis: (1) the Centers for Medicaid & Medicare Services (CMS) Minimum Data Set 3.0 (MDS), (2) the Medicare Provider and Analysis Review (MEDPAR), (3) the Master Beneficiary Summary File (MBSF), and (4) LTCFocus.^21^ The MDS is a standardized screening and assessment health status tool in CMS-certified NHs. MEDPAR is a CMS dataset of inpatient hospital claims. The MBSF is a CMS dataset describing all Medicare beneficiaries in a calendar year. LTCFocus is a publicly available, NH facility-level dataset derived from various datasets including the MDS, the Certification and Survey Provider Enhanced Reports systems, Medicare claims, and NH Compare.^21^

### Study Variables

The dependent variable of interest was PAHs. An expert CMS panel identified 16 PAH conditions as “potentially avoidable” using *International Statistical Classification of Diseases and Related Health Problems, Tenth Revision (ICD-10)* codes diagnosed upon hospital admission from an NH (ie, present on admission): chronic obstructive pulmonary disease and/or asthma, congestive heart failure, constipation, dehydration, hypertension, poor glycemic control, seizures, urinary tract infection, weight loss and/or malnutrition, altered mental status, anemia, diarrhea, falls and/or trauma, pneumonia, psychosis and/or agitation, or pressure injuries.^15,17^ MDS discharge assessments were used to identify hospitalizations. MEDPAR inpatient hospital records were linked to MDS NH discharges to confirm hospitalizations, and *ICD-10* codes identified PAHs. Resident characteristics may be affected by prior hospitalizations,^2^ therefore, we excluded any hospital admission prior to the index MDS (ie, first admission, annual, or quarterly) assessment to characterize patient acuity measures. Resident time at risk for PAHs varied by the timing of the index MDS assessment; thus, the dependent variable was the count of PAHs during the remainder of 2018 following the index MDS assessment.

The independent variables of interest were resident race and ethnicity. Race and ethnicity are self-reported in the MDS and databased in the MBSF. Residents with more than 1 race were considered multiracial. Mutually exclusive race and ethnicity categories were defined and analyzed: American Indian or Alaska Native, Asian, Black or African American, Hispanic, Native Hawaiian and Pacific Islander, White, and multiracial. Differences by race and ethnicity are socially constructed; these variables do not represent biological, cultural, or language differences between groups.^22,23^ The first MDS index assessment in 2018 was used to identify resident race and ethnicity, and if missing from MDS, was obtained from the MBSF using RTI race codes, which were created using an algorithm of US Census and geography.^24^ Residents with missing race or ethnicity were excluded from the analytic sample (n = 1004 [0.05%]).

Covariates included NH facility and resident characteristics. NH facility characteristics were obtained from LTCFocus and included size (ie, number of certified beds), chain status, and ownership of facility. Resident characteristics included: age, sex, dual eligibility (ie, Medicaid and Medicare coverage) in months, and chronic conditions, collected from the MBSF. Additionally, we controlled for resident time-at-risk exposure (ie, total time in NH for 2018 after the first index MDS assessment).

Severe cognitive impairment was measured on the cognitive function scale (CFS), which was designed for all NH residents and calculated by MDS assessments.^25^ The CFS is a valid tool for assessing resident cognitive assessment,^25^ regardless of English language proficiency.^26^ It is a 4-level scale: cognitively intact (CFS = 1), mildly impaired (CFS = 2), moderately impaired (CFS = 3), and severely impaired (CFS = 4). A resident was considered to have severe cognitive impairment (ie, CFS = 4) on all assessments after these criteria were first met from the first quarterly MDS assessment.^27^ Residents with a missing CFS score were excluded from the sample (n = 1592 [0.08%]).

### Statistical Analysis

Unadjusted and adjusted Poisson regression models with and without NH fixed effects were generated to estimate the incidence rate ratio (IRR) for PAHs across racial and ethnic categories. To account for arbitrary correlation structures among all observations within a US state, we calculated standard errors using block-bootstrap methods clustered at the state level.^28,29^

Four separate models were developed. Model 1 was an unadjusted model with race and ethnicity only. Model 2 was the unadjusted model with race and ethnicity and NH fixed effects. Models 1 and 2 provided information on unadjusted, crude PAH IRRs by race and ethnicity. Model 3 adjusted for resident and facility characteristics, and model 4 adjusted for resident characteristics and NH facility fixed effects. Adjusting for NH fixed effects allowed us to identify racial and ethnic differences that occurred within NHs. Comparing the models with and without NH fixed effects allowed us to determine the extent that differences were generated by variations within NHs vs between NHs. For example, if the IRR was 1.30 in model 1 and the corresponding within-NH IRR in model 2 was 1.15, then the 50% rate decrease ([15 / 30] × 100) indicates that half of the IRR difference is due to within-NH differences.

To investigate whether racial and ethnic differences in PAH rates differed by cognitive impairment, we estimated alternative versions of all models with an interaction term (race and ethnicity × severe cognitive impairment). Each hypothesis test was formally completed using a 2-tailed test with α = .05 as the statistical significance threshold. All statistical analyses were performed in Stata version 17 (StataCorp) from January to May 2022.

## Results

Among the 2 098 385 NH residents aged 65 and older from across the US included in the final sample, 7151 (0.3%) were American Indian or Alaska Native, 39 873 (1.9%) were Asian, 229 112 (10.9%) were Black or African American, 99 304 (4.7%) were Hispanic, 2785 (0.1%) were Native Hawaiian or Pacific Islander, 1 713 670 (81.7%) were White, and 6490 (0.3%) were multiracial; 1 355 143 (64.6%) were female; 128 997 (6.2%) had severe cognitive impairment; and the mean (SD) age was 81.8 (8.7) years. Asian residents were most likely (10.3%) to have severe cognitive impairment and White residents were least likely (5.6%) (Table 1).

**Table 1.** Characteristics of Nursing Home Residents

	No. (%)								*P* value^a^
	All residents	Nursing home resident race and ethnicity							
		American Indian or Alaska Native	Asian	Black or African American	Hispanic	Native Hawaiian or Pacific Islander	White	Multiracial	
No. (%)^b^	2 098 385 (100)	7151 (0.3)	39 873 (1.9)	229 112 (10.9)	99 304 (4.7)	2785 (0.1)	1 713 670 (81.7)	6490 (0.3)	<.001
Age, mean (SD), y	81.8 (8.7)	78.7 (8.3)	82.9 (8.6)	78.9 (8.8)	90.5 (8.5)	80.0 (8.5)	82.3 (8.7)	81.6 (8.6)	<.001
Sex									
Female	1 355 143 (64.6)	4297 (60.1)	25 233 (63.3)	139 135 (60.7)	58 632 (59.0)	1742 (62.5)	1 121 939 (65.5)	4164 (64.2)	<.001
Male	743 242 (35.4)	2854 (39.9)	14 640 (36.7)	89 977 (39.3)	40 672 (41.0)	1043 (37.5)	591 731 (34.5)	2326 (35.8)	
Dual-eligible months^c^									
0 mo	1 057 619 (50.4)	2138 (29.9)	11 483 (28.8)	69 545 (30.35)	22 577 (22.74)	1153 (41.40)	948,38 (55.34)	2340 (36.06)	<.001
1-11 mo	347 984 (16.6)	1491 (20.9)	6787 (17.0)	45 032 (19.66)	20 323 (20.47)	494 (17.74)	272 775 (15.92)	1082 (16.67)	
12 mo	692 783 (33.0)	3522 (49.3)	21 603 (54.2)	114 535 (49.99)	56 404 (56.80)	1138 (40.86)	492 513 (28.74)	3068 (47.27)	
CFS score									
Intact	993 544 (47.4)	3003 (42.0)	14 102 (35.4)	94 543 (41.3)	36 723 (37.0)	1251 (44.9)	841 194 (49.1)	2727 (42.0)	<.001
Mildly impaired	476 441 (22.7)	1812 (25.3)	8754 (22.0)	53 996 (23.6)	23 112 (23.3)	599 (21.5)	386 661 (22.6)	1507 (23.2)	
Moderately impaired	499 404 (23.8)	1892 (26.5)	12 927 (32.4)	62 221 (27.2)	30 568 (30.8)	709 (25.5)	389 356 (22.7)	1731 (26.7)	
Severely impaired	128 997 (6.2)	444 (6.2)	4090 (10.3)	18 352 (8.0)	8901 (9.0)	226 (8.1)	96 459 (5.6)	525 (8.1)	

Abbreviation: CFS, cognitive function score.

^a^ *P* values calculated using ANOVA and Pearson χ^2^ tests where applicable. Significance level is α = .05.

^b^ Represented in row percentages.

^c^ Represented in column percentages.

Table 2 presents models 1 and 2, the unadjusted analyses of the association between race and ethnicity, cognitive impairment status, and PAHs. In model 1, compared with White residents without severe cognitive impairment, Asian residents without severe cognitive impairment had lower PAH incidence (IRR, 0.81 [95% CI, 0.67-0.97]). American Indian or Alaska Native residents with severe cognitive impairment had a 49% greater PAH incidence compared with White residents with severe cognitive impairment (IRR, 1.49 [95% CI, 1.10-2.01]). Similar results were observed for Black or African American and Hispanic residents. When comparing models 1 and 2 for residents with severe cognitive impairment, Black or African American residents had 64% higher rates of PAH than White residents (model 1: IRR, 1.64 [95% CI, 1.48-1.81]), of which approximately 86% was contributed by within-NH effects (model 2: IRR, 1.55 [95% CI, 1.43-1.68]). Hispanic residents also had higher PAH rates than White residents, with approximately 80% of the total difference explained by within-NH effects (model 1: IRR, 1.45 [95% CI, 1.29-1.62]; model 2: IRR, 1.36 [95% CI, 1.25-1.48]). Overall, Black or African American residents consistently had the highest incidence of PAHs. Black or African American residents with severe cognitive impairment had a 64% greater incidence of a PAH compared with White residents (IRR, 1.64 [95% CI, 1.48-1.81]).

**Table 2.** Unadjusted Analyses of Potentially Avoidable Hospitalizations Incidence

Race and ethnicity^b^	PAH, IRR (95% CI)^a^	
	Model 1	Model 2 with NH fixed effects
Residents without severe cognitive impairment^c^		
American Indian or Alaska Native	1.10 (0.92-1.31)	1.02 (0.90-1.17)
Asian	0.81 (0.67-0.97)	0.88 (0.82-0.94)
Black or African American	1.19 (1.13-1.25)	1.10 (1.07-1.13)
Hispanic	1.04 (0.92-1.17)	0.99 (0.96-1.03)
Native Hawaiian or Pacific Islander	0.94 (0.78-1.12)	1.00 (0.86-1.15)
White	1 [Reference]	1 [Reference]
Multiracial	0.96 (0.79-1.17)	1.00 (0.88-1.13)
Residents with severe cognitive impairment		
American Indian or Alaska Native	1.49 (1.10-2.01)	1.53 (1.05-2.23)
Asian	1.19 (0.97-1.47)	1.29 (1.11-1.49)
Black or African American	1.64 (1.48-1.81)	1.55 (1.43-1.68)
Hispanic	1.45 (1.29-1.62)	1.36 (1.25-1.48)
Native Hawaiian or Pacific Islander	0.72 (0.48-1.07)	0.76 (0.52-1.10)
White	1 [Reference]	1 [Reference]
Multiracial	1.32 (0.88-1.96)	1.28 (0.88-1.87)

Abbreviations: IRR, incidence rate ratio; NH, nursing home; PAH, potentially avoidable hospitalization.

^a^ Unadjusted Poisson regression models with and without NH fixed effects were generated to estimate the IRR for PAHs across racial and ethnic categories. All standard errors were created using bootstrap methods with resampling cluster by state. Model 1 was an unadjusted model with race and ethnicity only. Model 2 was the unadjusted model with race and ethnicity and NH fixed effects. These models provided information on unadjusted, crude PAH incident rates by race and ethnicity. Significance level is α = .05.

^b^ Race and ethnicity, the independent variables of interest, are the interaction terms of race and ethnicity × severe cognitive impairment. They are presented by severe cognitive impairment status and race and ethnicity.

^c^ Severe cognitive impairment defined as cognitive function score = 4.

Multivariable analyses in models 3 and 4 (Table 3) showed that compared with White residents without severe cognitive impairment, Black or African American residents had 12% greater PAH incidence (model 3: IRR, 1.12 [95% CI, 1.08-1.17]). In model 3, among residents with severe cognitive impairment and compared with White residents, Black or African American (IRR, 1.48 [95% CI, 1.36-1.61]), and Hispanic (IRR, 1.28 [95% CI, 1.17-1.40]) residents had significantly greater PAH incidence. In the fixed-effects models (model 4), the highest PAH incidence was observed among Black or African American residents with a 48% higher incidence (IRR, 1.48 [95% CI, 1.36-1.60]). The fixed-effects model (model 4) comparison demonstrated that the within-NH variation explained approximately 100% of the differences in PAH incidence rates for Black or African American residents with severe cognitive impairment and approximately 96% for Hispanic residents with severe cognitive impairment.

**Table 3.** Multivariable Analyses of Potentially Avoidable Hospitalization Incidence

Race and ethnicity^b^	PAH, IRR (95% CI)^a^	
	Model 3	Model 4 with NH fixed effects
Residents without severe cognitive impairment^c^		
American Indian Alaska Native	1.01 (0.89-1.15)	0.98 (0.87-1.09)
Asian	0.90 (0.81-1.00)	0.98 (0.92-1.05)
Black African American	1.12 (1.08-1.17)	1.11 (1.09-1.14)
Hispanic	1.04 (0.93-1.15)	1.04 (1.01-1.08)
Native Hawaiian Pacific Islander	0.93 (0.82-1.06)	0.99 (0.88-1.12)
White	1 [Reference]	1 [Reference]
Multiracial	1.01 (0.88-1.17)	0.99 (0.87-1.13)
Residents with severe cognitive impairment		
American Indian Alaska Native	1.35 (0.99-1.85)	1.43 (0.99-2.05)
Asian	1.15 (0.99-1.34)	1.24 (1.06-1.45)
Black African American	1.48 (1.36-1.61)	1.48 (1.36-1.60)
Hispanic	1.28 (1.17-1.40)	1.27 (1.16-1.39)
Native Hawaiian Pacific Islander	0.73 (0.52-1.03)	0.78 (0.55-1.11)
White	1 [Reference]	1 [Reference]
Multiracial	1.28 (0.89-1.83)	1.25 (0.88-1.78)

Abbreviations: IRR, incidence rate ratio; NH, nursing home; PAH, potentially avoidable hospitalization.

^a^ Adjusted Poisson regression models with and without NH fixed effects were generated to estimate the IRR for PAHs across racial and ethnic categories. All standard errors were created using bootstrap methods with resampling cluster by state. Model 3 was the full model (with covariates adjusting for resident and facility characteristics) and model 4 was the full model and NH fixed effects. Covariates included nursing home facility fixed effects and (not shown): age, sex, dual eligibility status in months per resident, facility characteristics (total beds in facility, chain membership, for-profit status), resident chronic conditions, and exposure (ie, length of time at risk). Significance level is α = 0.05.

^b^ Race and ethnicity, the independent variables of interest, are the interaction terms of race and ethnicity × severe cognitive impairment. They are presented by severe cognitive impairment status and race and ethnicity.

^c^ Severe cognitive impairment defined as cognitive function score = 4.

## Discussion

In this cross-sectional study, historically marginalized NH residents with severe cognitive impairment had greater PAH incidence compared with their counterparts without severe cognitive impairment. Compared with White NH residents in unadjusted analyses accounting for NH fixed effects, American Indian or Alaska Native, Asian, Black or African American, and Hispanic residents with severe cognitive impairment had significantly greater PAH incidence compared with White residents. Increased prevalence persisted across all analyses for Black or African American and Hispanic residents.

Although disturbing, the greater PAH incidence observed among historically marginalized residents is not surprising. Regardless of cognitive impairment status, the greatest PAH incidence rates were consistently found among Black or African American residents. It is important to highlight that PAHs may result from neglectful care.^15^ Longstanding inequities in health care and unfair practices across structures and policies have resulted in cycles of disadvantage for historically marginalized populations.^30,31^ Historically marginalized residents with severe cognitive impairment have an even higher risk. They may have limited language abilities, which can potentially lead to worse care, poor recognition of distress, higher illness burden, and worsening symptoms that result in PAHs. Overall, the greater PAH incidence rates among historically marginalized residents were often explained by differences within NHs. These results suggest that regardless of which NH the resident resides in, American Indian or Alaska Native, Asian, Black or African American, and Hispanic residents with severe cognitive impairment have greater PAH incidence. Racial and ethnic inequities in NH care have been long documented, including poor pain management,^32^ poorer quality of life,^33,34^ and overall poor quality of care.^35,36,37^ NHs are often segregated, racial disparities are pervasive, and structural racism are all known factors that have led to these inequities in NH care.^18^

Our findings suggest that early identification and monitoring of cognitive impairment may be crucial to improving EOL care outcomes, particularly for American Indian or Alaska Native, Black or African American, and Hispanic residents for 3 reasons. First, we found that residents without severe cognitive impairment had lower PAH incidence compared with residents with severe cognitive impairment. Early identification of cognitive impairment can reduce PAHs given the known associations between cognitive decline and hospitalizations.^2^ Particularly for residents with cognitive impairment, monitoring, early identification, and timely follow-up to prevent or manage the condition in the NH as medically appropriate should occur to avoid escalation of the underlying condition and unnecessary hospitalization. Second, given that American Indian or Alaska Native, Asian, Black or African American, and Hispanic residents with severe cognitive impairment had higher incidence rates of PAHs compared with White residents, careful monitoring of cognitive function before and after hospitalizations may prevent further escalation. Notably, we found that Asian residents were more likely to have cognitive impairment, which is inconsistent with some community-based samples.^38^ However, comparisons with prior work are limited because of differences in sample characteristics (eg, included small Japanese and Korean American populations)^38^ while we had a representative nationwide sample. Third, finding that historically marginalized NH residents with severe cognitive impairment have greater PAH incidence provides evidence for future research interventions to reduce EOL PAHs and achieve health equity for historically marginalized NH residents. Residents with severe cognitive impairment, as with others with serious illness, should have EOL care that upholds resident and family values and wishes, and efforts to describe and document preferences and implement goal concordant care should be executed before cognitive and physical decline.^39^

Given the high rate of cognitive impairment among NH residents, PAHs are naturally more common for individuals with cognitive impairment, which highlights the importance of our findings. We found that American Indian or Alaska Native, Asian, Black or African American, and Hispanic residents had greater PAH incidence compared with White NH residents. Black or African American and Hispanic residents often have delayed diagnosis,^40^ and cognitive impairment prevalence and diagnosis has been understudied among the American Indian or Alaska Native population.^41^ Unfortunately, this might imply that more residents of these populations have severe cognitive impairment than are reported and, as a result, actual incidence rates for PAHs may be underestimated.

The EOL experience is individualized and the primary goal for better outcomes is to provide person-centered, family-oriented care, and provide comfort.^42^ PAHs are not consistent with these goals. An important component for EOL care in NHs is the integration of palliative care. Palliative care is medical care for individuals with serious illness and their families, such as NH residents, focused on improving quality of life through relief of symptoms and stress related to illness, regardless of prognosis.^43^ Palliative care has the potential to reduce inequities in NH care, particularly at the EOL for historically marginalized NH residents, by providing patient-centered care. The integration of palliative care and advanced dementia care has been shown to improve symptom management for persons with dementia in community settings.^44,45^ In a study of 31 NHs, palliative care consultations for residents with moderate to severe dementia reduced hospitalizations for residents in the days prior to death.^46^ Recent national initiatives from the CMS Innovation and the Medicare-Medicaid Coordination Office have aimed to reduce NH PAHs, but none were specific to palliative care.^47^ These initiatives are often focused on equality, which provides the same resources for all NHs, instead of focusing on equity, which prioritizes need.^48^ Indeed, wide variation in NH palliative care services nationwide has been identified,^49^ and disturbingly, NHs with increased concentration of Black or African American or Hispanic residents have fewer palliative care services.^31^ Given our results identifying differences both between NHs and within NHs, further examination is warranted regarding the association of PAHs and palliative care services for historically marginalized NH residents.

### Limitations

This study has limitations that must be acknowledged. First, the nonrandomized design prevents causal interpretations of the results. Second, the MDS data are subject to self-reporting biases from staff. However, these data are federally regulated and found to be both valid and reliable.^50^ Third, although we controlled for the resident’s time of residence in the NH during the year 2018 (ie, time at risk), our ability to account for the total resident time at risk was limited, which would require longitudinal data. However, given the validity and reliability of the nationwide datasets used, we are confident that these findings accurately represent racial and ethnic PAH incidence rates for historically marginalized NH residents compared with White NH residents.

## Conclusions

This study has important implications for NH residents with severe cognitive impairment. Higher PAH incidence rates were observed across all historically marginalized residents compared with White residents, and especially for American Indian or Alaska Native, Black or African American, and Hispanic residents with and without severe cognitive impairment, despite national initiatives to reduce these hospitalizations. Although our findings suggest that the PAH incidence differences were explained by both between-NH factors and within-NH factors, a large fraction of the total difference was explained by within-NH differences, which may be related to a combination of experiences with racism and discrimination. Future research should examine how palliative care can reduce PAHs, specifically for historically marginalized residents with severe cognitive impairment.

## Notes

Supplement.
Data Sharing Statement
