# Supplementary material for: Potentially Avoidable Hospitalizations Among Historically Marginalized Nursing Home Residents
Source: JAMA Netw Open. 2024 May 2;7(5):e249312. doi: 10.1001/jamanetworkopen.2024.9312 (PMC11066698; doi:10.1001/jamanetworkopen.2024.9312)
Supplement: Supplement. — Data Sharing Statement [file jamanetwopen-e249312-s001.pdf]

Data Sharing Statement

Estrada. Potentially Avoidable Hospitalizations Among Historically Marginalized Nursing Home Residents. *JAMA Netw Open*. Published May 02, 2024.
doi:10.1001/jamanetworkopen.2024.9312

Data
Data available: No

Additional Information
Explanation for why data not available: This study used data from the Centers for Medicare & Medicaid Services and in congruence with our Data Use Agreement with CMS, we are not allowed to share these data.
